# lncRNA TUG1 promotes proliferation and differentiation of osteoblasts by regulating the miR-545-3p/CNR2 axis

**DOI:** 10.1590/1414-431X20209798

**Published:** 2020-10-07

**Authors:** Ruizheng Hao, Bin Wang, Hui Wang, Yongxin Huo, Yang Lu

**Affiliations:** 1 Department of Hand Surgery, The Second Hospital of Tangshan, Tangshan, Hebei, China; 2 Department of Orthopedics, First Hospital of Qinhuangdao, Qinhuangdao, Hebei, China

**Keywords:** TUG1, miR-545-3p, CNR2, Osteoblasts, Proliferation, Differentiation

## Abstract

Osteoblast differentiation is an effective way to promote bone formation. Long non-coding RNA taurine upregulated 1 (TUG1) has been identified as a crucial modulator of multiple biological processes. This study was designed to investigate the function of TUG1 in the proliferation and differentiation of osteoblast precursor cells hFOB1.19. In this study, we found that TUG1 promoted hFOB1.19 cell proliferation, while TUG1 knockdown hindered cell proliferation. TUG1 and cannabinoid receptor 2 (CNR2) were upregulated, while miR-545-3p was down-regulated in hFOB1.19 cells undergoing osteoblastic differentiation. TUG1 induced osteoblast differentiation by increasing alkaline phosphatase (ALP) activity and the expression of osteoblastic differentiation markers. TUG1 was a sponge of miR-545-3p and regulated osteoblastic differentiation by modulating miR-545-3p. Moreover, miR-545-3p directly targeted CNR2 and restored the effect of CNR2 on osteoblastic differentiation. In conclusion, TUG1 accelerated the proliferation and differentiation of osteoblasts by sponging miR-545-3p and increasing CNR2 expression, which might provide a new biomarker for bone diseases.

## Introduction

Bone is an organ dynamically regulated by osteoblasts and osteoclasts, which emerge as crucial regulators in bone turnover (1). The major responsibility of osteoblasts is bone formation, while the primary function of osteoclasts is bone resorption (2). Unbalance of osteoblast and osteoclast functions causes several bone metabolic diseases, including osteoporosis (3). Osteogenic differentiation is an orderly process in which mesenchymal stem cells (MSCs) are transformed into osteoblasts (4). Accumulating evidence has shown that the induction of osteogenic differentiation is a vital therapeutic strategy for bone diseases.

Long non-coding RNAs (lncRNAs) are a type of non-coding RNAs (ncRNAs) longer than 200 nucleotides. Numerous studies have shown that lncRNAs are abnormally expressed in many diseases and participate in the occurrence and development of tumors (5). lncRNA taurine upregulated 1 (TUG1) has been documented to be aberrantly expressed in many cancers (6). For example, TUG1 contributed to cell proliferation and metastasis in melanoma by sponging microRNA-29c-3p and upregulating RGS1 (regulator of G-protein signaling 1) (7). TUG1 recruited microRNA-212-3p from FOXA1 (Forkhead box A1) to expedite osteosarcoma progression (8). Besides, TUG1 diminished cisplatin resistance in triple negative breast cancer by binding to microRNA-197 (9). In osteoblast differentiation, TUG1 silencing restrained osteoblast proliferation and differentiation via inactivating the Wnt/β-catenin pathway (10). Nevertheless, the precise mechanism of TUG1 in osteoblast differentiation remains poorly understood.

MicroRNAs (miRNAs) are highly conserved ncRNAs composed of 18-25 nucleotides. Emerging evidence has validated that miRNAs exert a crucial regulatory role in osteoblast differentiation and osteoclast-mediated bone resorption by repressing mRNA translation (11). For instance, inhibition of miR-451a facilitated osteogenic differentiation and impeded bone loss in osteoporosis by regulating Bmp6 expression (12). Down-regulation of miR-92b-5p regulated ICAM-1 (intercellular adhesion molecule 1) expression to block osteogenic differentiation stimulated by melatonin in bone marrow mesenchymal stem cells (BMSCs) (13). In addition, lncRNAs can serve as miRNA sponges to regulate gene expression (14). In the present research, bioinformatics analysis revealed that TUG1 might be a decoy for miR-545-3p. Previous research found that miR-545-3p hindered osteogenic differentiation (15). However, the relationship between TUG1 and miR-545-3p in osteogenic differentiation remains unclear.

Moreover, cannabinoid receptor 2 (CNR2) might be a target of miR-545-3p based on bioinformatics analysis. CNR2 is implicated in cancer, bone metabolism, and pain perception (16). CNR2 is more implicated in the physiological modulation of the skeleton compared to other pathological processes of the central nervous system (17,18). In renal cell carcinoma, CNR2 knockdown curbed tumor progression (19). In osteoporosis, upregulation of CNR2 accelerated the osteogenic differentiation of BMSCs (20).

In this research, we verified the role of TUG1 in hFOB1.19 cell proliferation and differentiation. More importantly, we explored the potential mechanisms of TUG1 in osteogenic differentiation.

## Material and Methods

### Cell culture

Human fetal osteoblastic cell line hFOB1.19 was commercially acquired from American Type Culture Collection (ATCC, USA). Cells were maintained in Dulbecco's modified Eagle medium (DMEM)/Ham's F-12 medium (1:1) (Gibco, USA) supplemented with 10% fetal bovine serum (FBS; Gibco) and 0.3 mg/mL geneticin (G-418; Solarbio, China). For osteoblastic differentiation, hFOB1.19 cells were cultured in osteogenic medium (OM; Gibco, USA) for different time periods (0, 1, 4, 7, 14, or 21 days). Cells were seeded in an incubator with 5% CO_2_ at 34°C for proliferation and at 39°C for differentiation.

### Cell transfection

TUG1 overexpression vector, CNR2 overexpression vector, the empty overexpression vector, small interference RNA (siRNA) against TUG1 (si-TUG1), siRNA targeting CNR2 (si-CNR2), siRNA negative control (si-NC), miR-545-3p mimic (miR-545-3p), the scramble control (NC), miR-545-3p inhibitor (anti-miR-545-3p), and the inhibitor control (anti-NC) were purchased from GenePharma (China). When cell confluence reached ∼70%, plasmids and oligonucleotides were transfected into hFOB1.19 cells using Lipofectamine 3000 (Invitrogen, USA).

### Quantitative real-time polymerase chain reaction (qRT-PCR)

After RNA extraction using Trizol reagent (Invitrogen), RNA was reverse-transcribed using the Prime script RT master mix (Takara, China) or miScript reverse transcription kit (Qiagen, Germany). qRT-PCR was performed using SYBR Green PCR master mix (Takara). The expression of TUG1 and CNR2 was normalized by β-actin. MiR-545-3p expression was normalized by U6. RNA levels were calculated using the 2^-ΔΔCt^ method. The reaction procedure included 95°C for 10 min, followed by 40 cycles of 95°C for 10 s, 60°C for 10 s, and 72°C for 30 s. The primers included: TUG1-F: 5′-CTGAAGAAAGGCAACATC-3′, TUG1-R: 5′-GTAGGCTACTACAGGATTTG-3′; miR-545-3p-F: 5′-TGGCTCAGTTCAGCAGGAAC-3′, miR-545-3p-R: 5′-TGGTGTCGTGGAGTCG-3′; CNR2-F: 5′-GGGTGACAGAGATAGCCAATGG-3′, CNR2-R: 5′-TGAACAGGTATGAGGGCTTCC-3′; β-actin-F: 5′-GTCACCGGAGTCCATCACGAT-3′, β-actin-R: 5′-TCACCAACTGGGACGACATG-3′; U6-F: 5′-CTCGCTTCGGCAGCACA-3′, U6-R: 5′-AACGCTTCACGAATTTGCGT-3′.

### Cell counting kit-8 (CCK-8) assay

Transfected hFOB1.19 cells (5×10^3^) were plated into 96-well plates. Then, the cells were cultured for 24, 48, or 72 h. After discarding the cell supernatant, 100 μL complete medium containing 10 μL CCK-8 solution (Beyotime, China) was added to each well at indicated time points. After incubation for 3 h, the absorbance was monitored at 450 nm using Varioskan™ LUX multimode microplate reader (Thermo Fisher Scientific, USA).

### Western blot assay

Cells were lysed using RIPA buffer (Solarbio) and then subjected to sodium dodecyl sulfate polyacrylamide gel electrophoresis (SDS-PAGE) to separate proteins. Subsequently, the proteins were transferred onto polyvinylidene fluoride membranes (Millipore, USA). The membranes were blocked with 5% non-fat milk for 2 h, and then incubated with primary antibodies against proliferating cell nuclear antigen (PCNA) (ab18197, Abcam, UK), CNR2 (ab3561, Abcam), ALP (ab83259, Abcam), runt-related transcription factor 2 (RUNX2) (ab23981, Abcam), osteocalcin (OCN) (ab93876, Abcam), osteopontin (OPN) (ab8448, Abcam), and β-actin (ab8227, Abcam). Next, the membranes were probed with the corresponding secondary antibody (ab7090, Abcam). Subsequently, the signal intensity was detected by the enhanced chemiluminescence system (Qiagen).

### Alkaline phosphatase (ALP) activity determination

ALP activity, a hallmark enzyme of mature osteoblasts, was examined using the ALP activity colorimetric assay kit (BioViSion, USA). First, the cells were washed twice with PBS and then extracted with RIPA buffer (Solarbio). The optical density at 405 nm was measured using Varioskan™ LUX multimode microplate reader (Thermo Fisher Scientific).

### Dual-luciferase reporter assay

The sequences of TUG1 or CNR2 3′ untranslated region (UTR) containing wild-type or mutant binding site of miR-545-3p were inserted into pmirGLO vector (Promega, USA) to form TUG1-wt (wild type), TUG1-mut (mutant), CNR2-wt, or CNR2-mut. Then, the corresponding vector and miR-545-3p mimic or NC were co-transfected into hFOB1.19 cells. The Dual-luciferase reporter kit (Promega) was utilized to measure luciferase activity.

### RNA immunoprecipitation (RIP) assay

RIP assay was performed using Magna RNA immunoprecipitation kit (Millipore). In brief, hFOB1.19 cells were transfected with miR-545-3p or NC. After collecting cell lysates, they were incubated with magnetic beads containing anti-Ago2 or anti-IgG. Finally, the enrichment of TUG1 and miR-545-3p was measured by qRT-PCR.

### Statistical analysis

Graphpad Prism 7.0 software (USA) was utilized to analyze the data. Data are reported as means±SD with three independent experiments. The difference was analyzed by Student's *t*-test and one-way analysis of variance. P<0.05 was considered statistically significant.

## Results

### lncRNA TUG1 promoted hFOB1.19 cell proliferation

**Figure 1:**
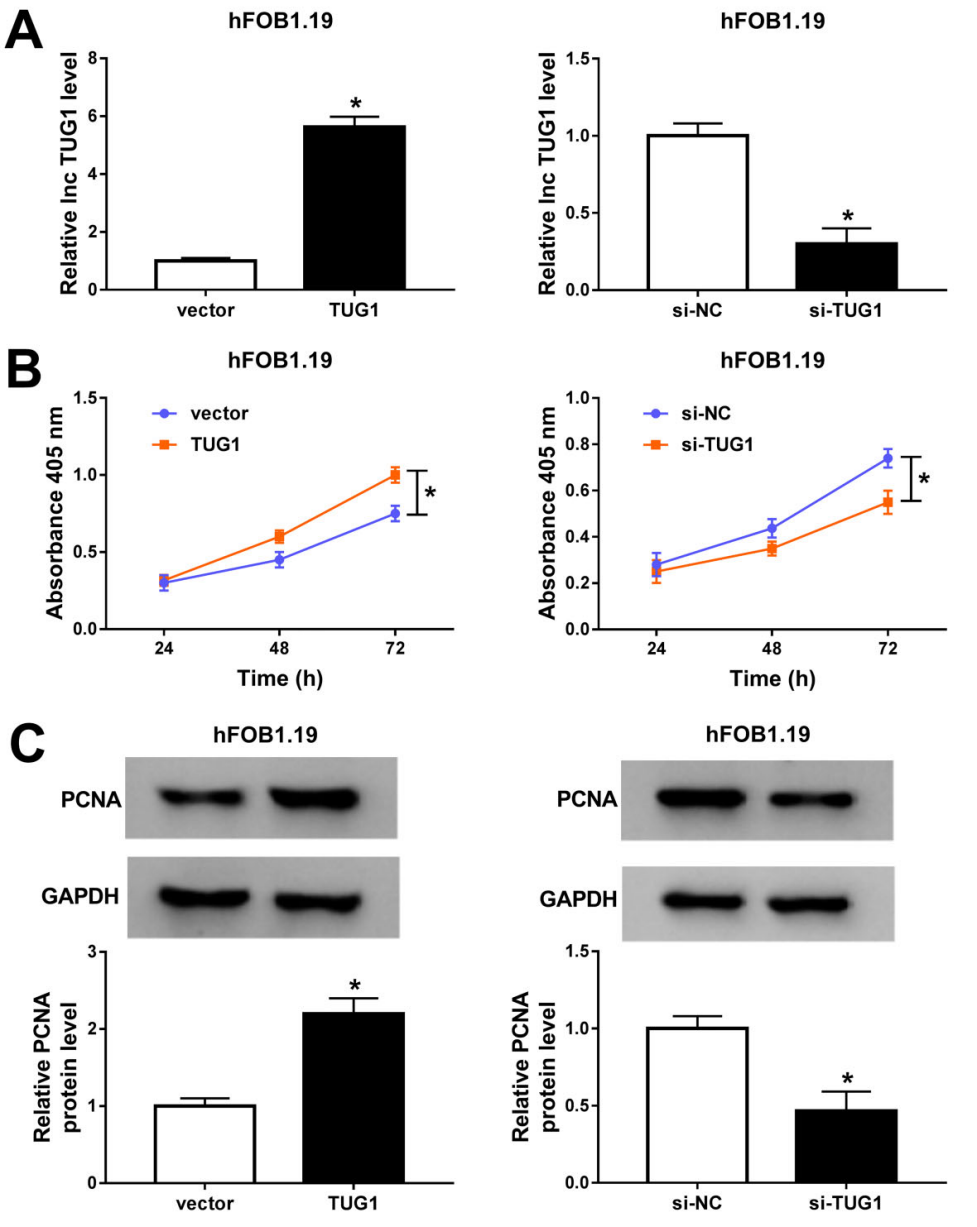
Long non-coding RNA TUG1 promoted hFOB1.19 cell proliferation. **A**-**C**, TUG1 was overexpressed or knocked down in hFOB1.19 cells after introduction of TUG1 or si-TUG1. **A**, The expression of TUG1 was determined by qRT-PCR. **B**, Cell viability was detected by CCK-8 assay. **C**, The expression of PCNA was measured by western blot assay. Data are reported as means±SD of three independent experiments. *P<0.05 (Student's *t*-test).

To investigate the effect of TUG1 on cell proliferation, hFOB1.19 cells were transfected with TUG1 or si-TUG1. First, transfection efficiency was evaluated using qRT-PCR. TUG1 expression in the TUG1 group was significantly higher than that in the vector group, and TUG1 expression in the si-TUG1 group was significantly lower than that in the si-NC group (Figure 1A). In addition, CCK-8 analysis showed that overexpression of TUG1 significantly expedited the viability of hFOB1.19 cells, while down-regulation of TUG1 suppressed hFOB1.19 cell viability (Figure 1B). Moreover, western blot assay revealed that PCNA expression was elevated after transfection with TUG1, whereas PCNA expression was decreased after the introduction of si-TUG1 (Figure 1C). These data indicated that TUG1 promoted hFOB1.19 cell proliferation.

### TUG1 and CNR2 were upregulated, while miR-545-3p was down-regulated in osteogenic differentiated hFOB1.19 cells

**Figure 2:**
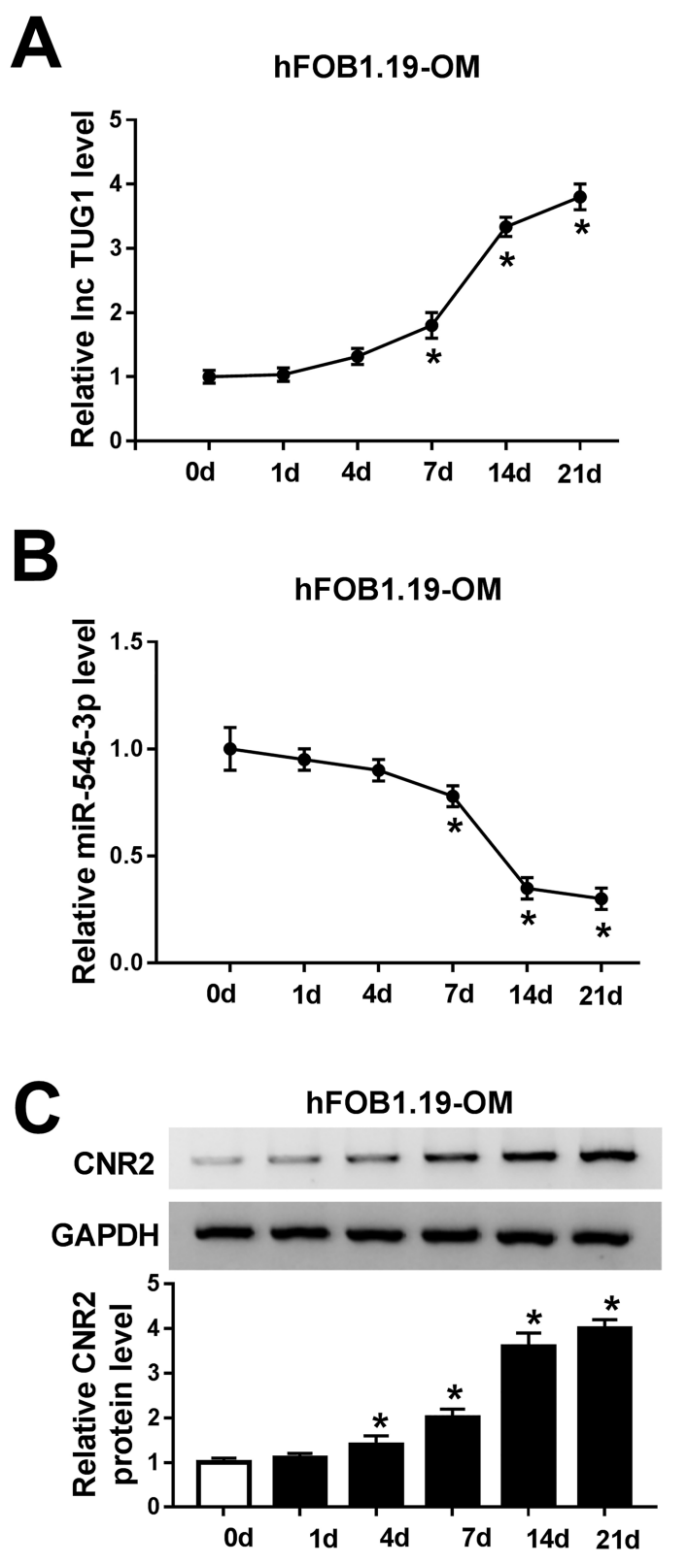
TUG1 and CNR2 were upregulated, while miR-545-3p was down-regulated in osteogenic differentiated hFOB1.19 cells. The hFOB1.19 cells were incubated in osteogenic medium (OM) for 0, 1, 4, 7, 14, or 21 d. **A** and **B**, TUG1 and miR-545-3p expression was detected by qRT-PCR. **C**, CNR2 protein level was examined by western blot assay. Data are reported as means±SD of three independent experiments. *P<0.05 (ANOVA).

TUG1 expression was significantly increased in hFOB1.19 cells cultured with OM in a time-dependent manner (Figure 2A). qRT-PCR suggested a significant reduction in miR-545-3p expression at 7, 14, and 21 days in hFOB1.19 cells stimulated with OM (Figure 2B). Furthermore, western blot assay indicated a marked increase in CNR2 expression at 4, 7, 14, and 21 days in hFOB1.19 cells cultured with OM (Figure 2C). These data suggested that TUG1 might play a vital role in osteogenic differentiation.

### ALP activity and marker expression were increased during osteogenesis

**Figure 3:**
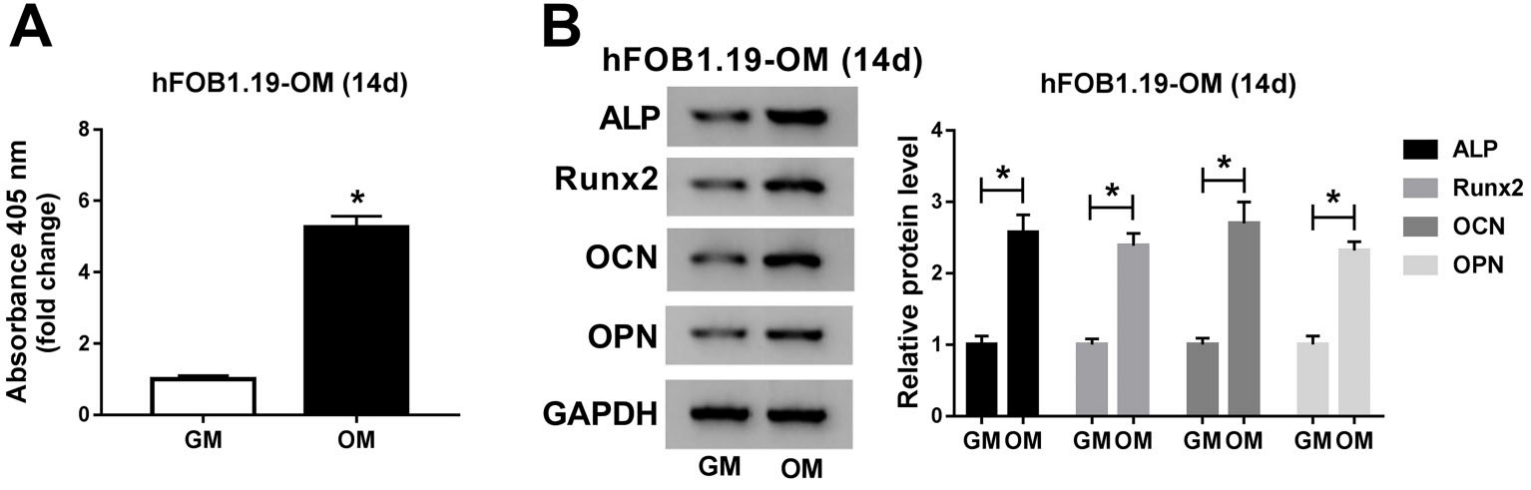
ALP activity and marker expression were increased during osteogenesis. **A**, The activity of ALP was determined in hFOB1.19 cells cultured with growth medium (GM) or osteogenic medium (OM) (14d). **B**, The expression of osteogenic differentiation markers was determined by western blot assay. Data are reported as means±SD of three independent experiments. *P<0.05 (*t*-test).

ALP activity was increased in hFOB1.19-OM cells compared with hFOB1.19-control cells (Figure 3A). Similarly, the levels of osteogenic-related proteins (ALP, Runx2, OCN, and OPN) were significantly higher in hFOB1.19-OM cells than that in hFOB1.19-control cells (Figure 3B).

### TUG1 facilitated hFOB1.19 cell differentiation

**Figure 4:**
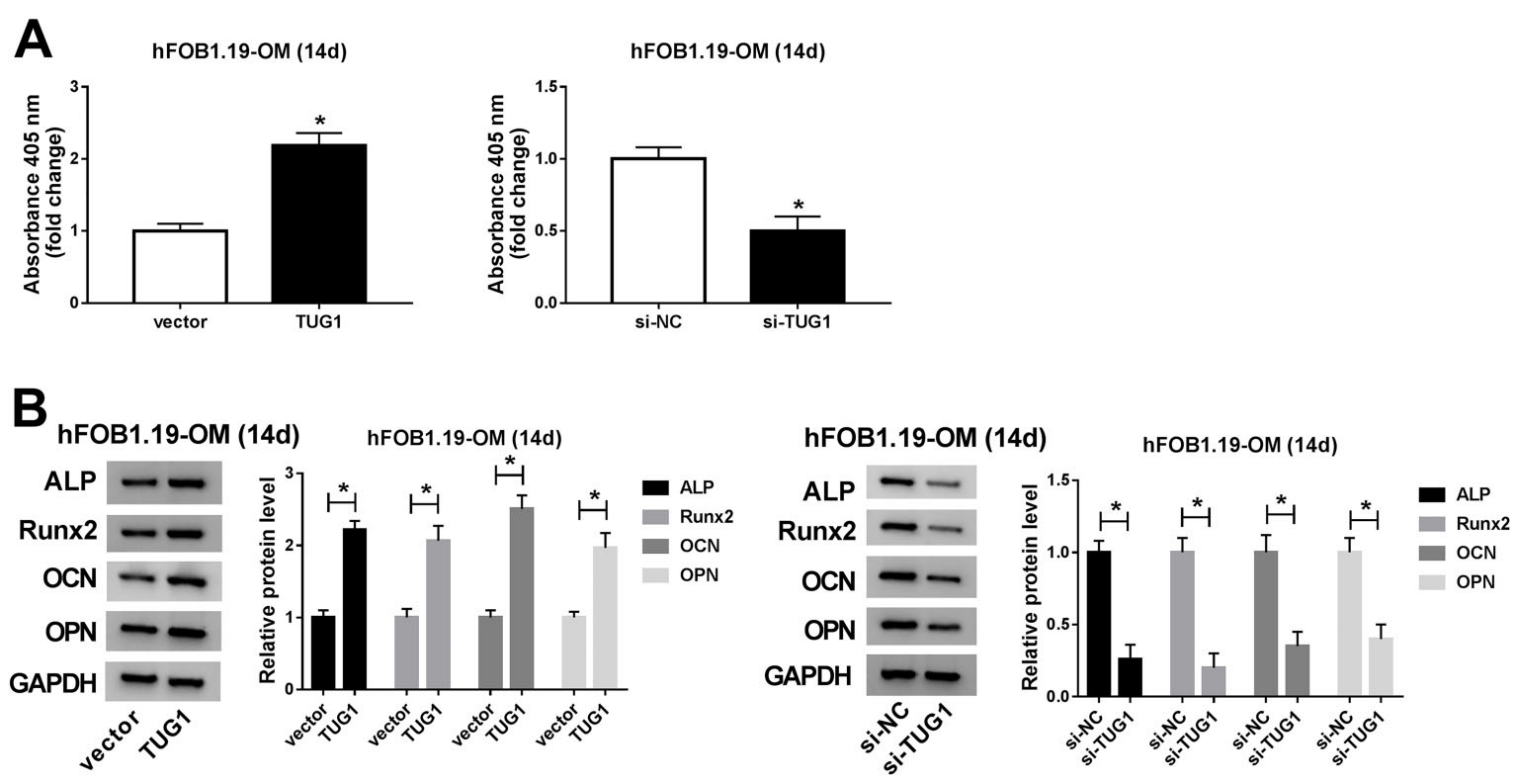
TUG1 facilitated hFOB1.19 cell differentiation. **A**, The activity of ALP in osteogenic differentiated hFOB1.19 cells was detected after TUG1 was upregulated or down-regulated. **B**, The protein levels of ALP, Runx2, osteocalcin (OCN), and osteopontin (OPN) in osteogenic differentiated hFOB1.19 cells were measured after transfection with TUG1 or si-TUG1. Data are reported as means±SD of three independent experiments. *P<0.05 (*t*-test).

ALP activity was increased in hFOB1.19-OM cells by overexpressing TUG1 relative to the vector group, whereas knockdown of TUG1 restrained the activity of ALP (Figure 4A). Moreover, the protein levels of ALP, Runx2, OCN, and OPN were significantly increased in hFOB1.19-OM cells transfected with TUG1 compared with the vector group, while the levels were decreased in osteogenic differentiated hFOB1.19 cells introduced with si-TUG1 (Figure 4B). These data indicated that TUG1 facilitated hFOB1.19 cell differentiation.

### TUG1 directly targeted miR-545-3p

**Figure 5:**
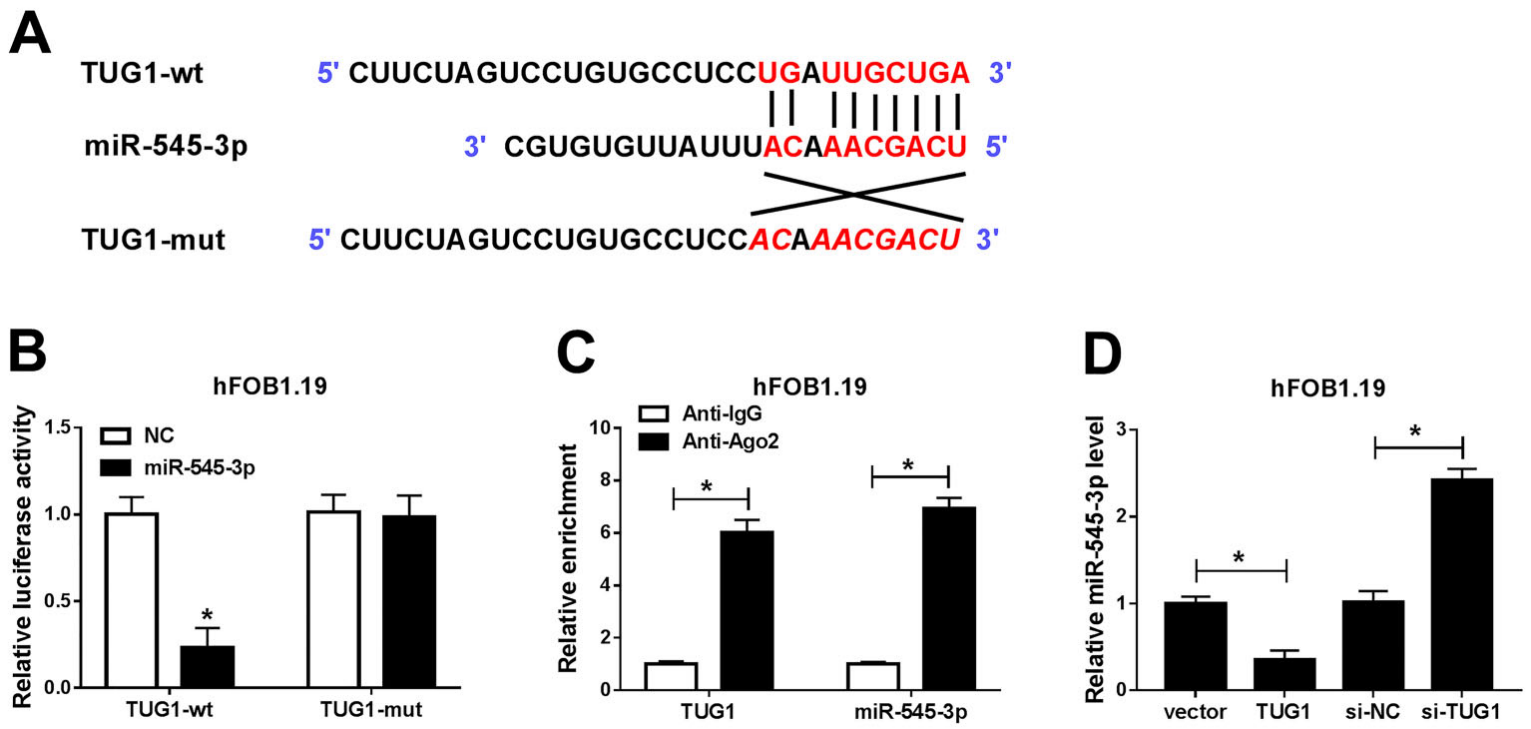
TUG1 directly targeted miR-545-3p. **A**, The predicted binding sites between TUG1 and miR-545-3p are shown. **B**, Luciferase activity was detected in hFOB1.19 cells co-transfected with TUG1-wt (wild type) or TUG1-mut (mutated) and miR-545-3p or negative control (NC). **C**, RIP assay was used to measure the enrichment of TUG1 and miR-545-3p in the immunoprecipitation complex. **D**, Expression of miR-545-3p was examined in hFOB1.19 cells transfected with vector, TUG1, si-NC, or si-TUG1. Data are reported as means±SD of three independent experiments. *P<0.05 (*t*-test).

LncBase Predicted v.2 online database (http://carolina.imis.athena-innovation.gr/diana_tools/web/index.php?r=lncbasev2/index-predicted) predicted that TUG1 contained the complementary binding sites of miR-545-3p (Figure 5A). Dual-luciferase reporter assay disclosed that the luciferase activity of TUG1-wt reporter was significantly decreased after transfection with miR-545-3p mimic, but the luciferase activity of TUG1-mut reporter was not affected when the binding sites were mutated (Figure 5B). Furthermore, RIP assay was performed to verify whether miR-545-3p was a target of TUG1. The results showed that TUG1 and miR-545-3p were enriched in Ago2 antibody complex compared with the anti-IgG group (Figure 5C). In addition, miR-545-3p expression was detected in hFOB1.19 cells introduced with vector, TUG1, si-NC, or si-TUG1. The results showed that upregulation of TUG1 resulted in a distinct decrease in miR-545-3p expression, while down-regulation of TUG1 induced a significant increase in miR-545-3p expression (Figure 5D). These data demonstrated that miR-545-3p was a direct target of TUG1 in hFOB1.19 cells.

### TUG1 restored the effect of miR-545-3p on osteogenic differentiation

**Figure 6:**
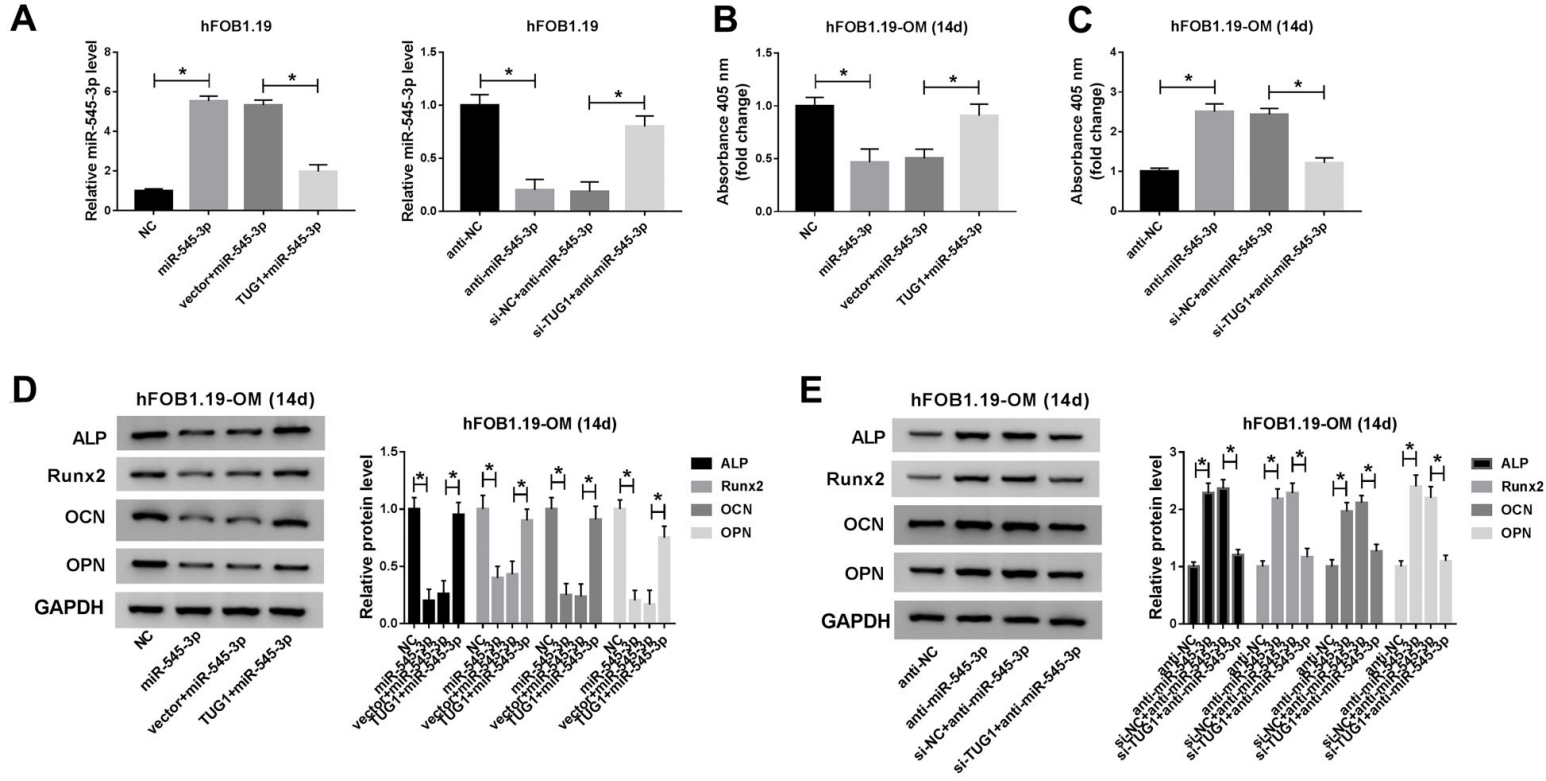
TUG1 restored the effect of miR-545-3p on osteogenic differentiation. **A**, Expression of miR-545-3p was measured in hFOB1.19 cells transfected with negative control (NC), miR-545-3p, vector+miR-545-3p, or TUG1+miR-545-3p. Also, miR-545-3p expression was tested in hFOB1.19 cells transfected with anti-NC, anti-miR-545-3p, si-NC+anti-miR-545-3p, or si-TUG1+anti-miR-545-3p. **B**, The activity of ALP was detected in osteogenic differentiated hFOB1.19 cells following miR-545-3p upregulation and/or TUG1 upregulation. **C**, ALP activity was examined in osteogenic differentiated hFOB1.19 cells after miR-545-3p down-regulation and/or TUG1 down-regulation. **D**, Protein levels of ALP, Runx2, osteocalcin (OCN), and osteopontin (OPN) were analyzed in osteogenic differentiated hFOB1.19 cells transfected with miR-545-3p and/or TUG1. **E**, The levels of ALP, Runx2, OCN, and OPN were tested in osteogenic differentiated hFOB1.19 cells introduced with anti-miR-545-3p and/or si-TUG1 by western blot assay. Data are reported as means±SD of three independent experiments. *P<0.05 (ANOVA).

To investigate the role of miR-545-3p in TUG1-mediated osteogenic differentiation, ALP activity and osteoblastic differentiation markers were detected in osteogenic differentiated hFOB1.19 cells after transfection. The results of qRT-PCR showed that co-transfection of miR-545-3p and TUG1 recovered the increase in miR-545-3p expression induced by transfection of miR-545-3p, and the reduction in miR-545-3p expression triggered by inhibition of miR-545-3p was restored after introduction of anti-miR-545-3p and si-TUG1 (Figure 6A). Moreover, the activity of ALP was significantly restrained in miR-545-3p-treated osteogenic differentiated hFOB1.19 cells, while TUG1 upregulation abrogated this effect (Figure 6B). Similarly, inhibition of miR-545-3p increased ALP activity, which was weakened by TUG1 knockdown (Figure 6C). In addition, overexpression of miR-545-3p resulted in a decrease in the levels of ALP, Runx2, OCN, and OPN, whereas the levels were reverted by reintroduction of TUG1 (Figure 6D). Consistently, transfection with anti-miR-545-3p significantly increased the expression of ALP, Runx2, OCN, and OPN, while the effect was relieved after transfection with si-TUG1 (Figure 6E). These results showed that TUG1 restored the inhibitory effect of miR-545-3p on osteogenic differentiation in hFOB1.19 cells.

### CNR2 was a target of miR-545-3p

**Figure 7:**
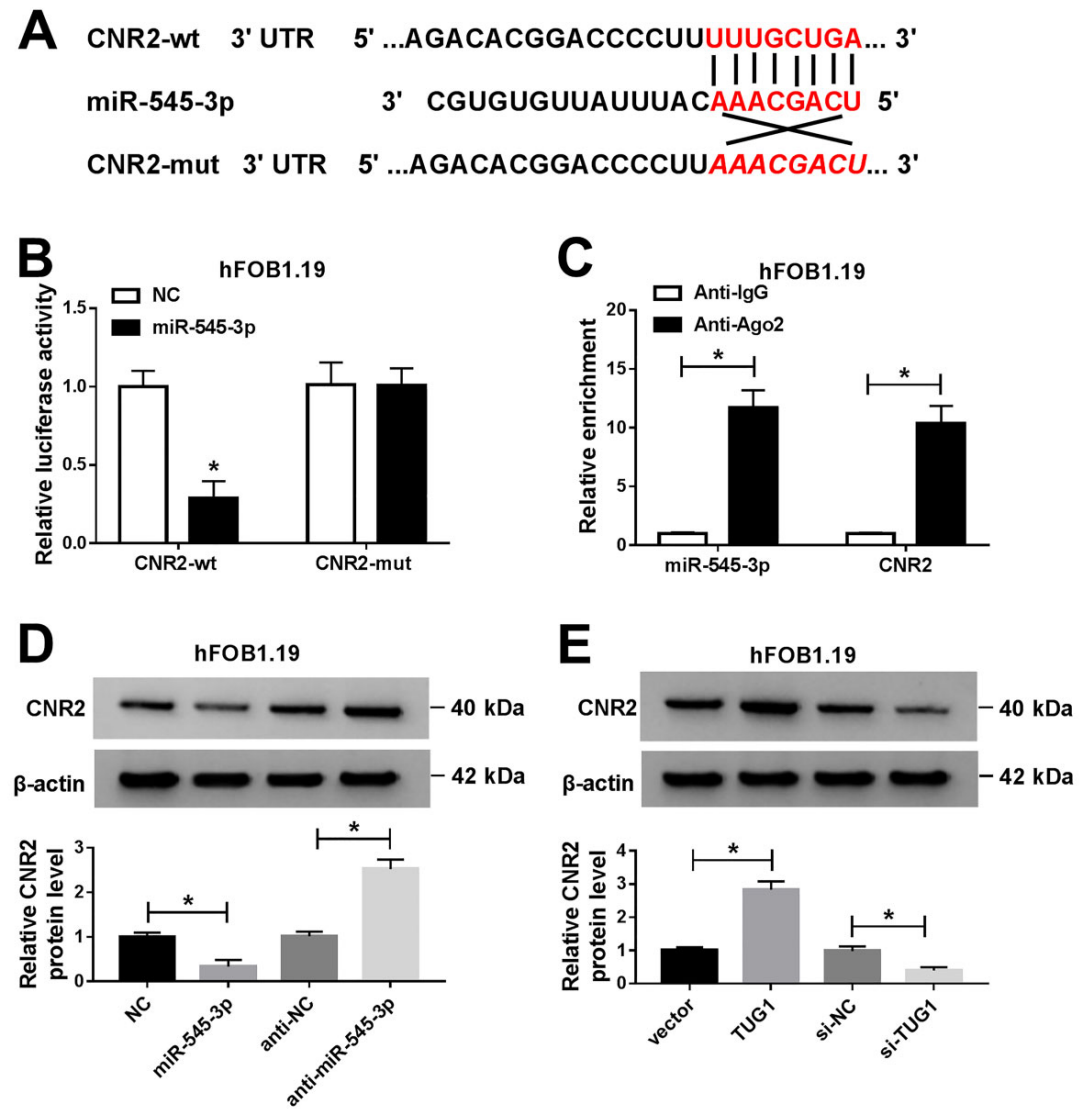
CNR2 was a target of miR-545-3p. **A**, The putative binding sites for miR-545-3p and CNR2 3'UTR are shown. **B**, Dual-luciferase reporter assay was conducted to confirm the relationship between miR-545-3p and CNR2. **C**, RIP assay was utilized to validate the correlation between miR-545-3p and CNR2. **D**, The protein expression of CNR2 was examined in hFOB1.19 cells transfected with negative control (NC), miR-545-3p, anti-NC, or anti-miR-545-3p. **E**, After transfection with vector, TUG1, si-NC, or si-TUG1, CNR2 expression was tested by western blot assay. Data are reported as means±SD of three independent experiments. *P<0.05 (*t*-test).

TargetScan online database (http://www.targetscan.org/vert_72/) predicted that miR-545-3p and CNR2 3′UTR had putative binding sites (Figure 7A). Dual-luciferase reporter assay showed that mature miR-545-3p significantly blocked the luciferase activity of CNR2-wt reporter (Figure 7B). RIP assay was used to further confirm whether miR-545-3p targeted CNR2, and the results revealed that miR-545-3p and CNR2 were enriched in anti-Ago2 complex (Figure 7C). Furthermore, the protein level of CNR2 was significantly decreased in hFOB1.19 cells transfected with miR-545-3p compared to the NC group, and the protein level of CNR2 was significantly increased in hFOB1.19 cells introduced with anti-miR-545-3p relative to the anti-NC group (Figure 7D). Besides, upregulation of TUG1 significantly increased CNR2 protein expression, and depletion of TUG1 reduced CNR2 protein expression (Figure 7E). These data indicated that CNR2 was a target of miR-545-3p in hFOB1.19 cells.

### miR-545-3p inversed the effect of CNR2 on osteogenic differentiation

**Figure 8:**
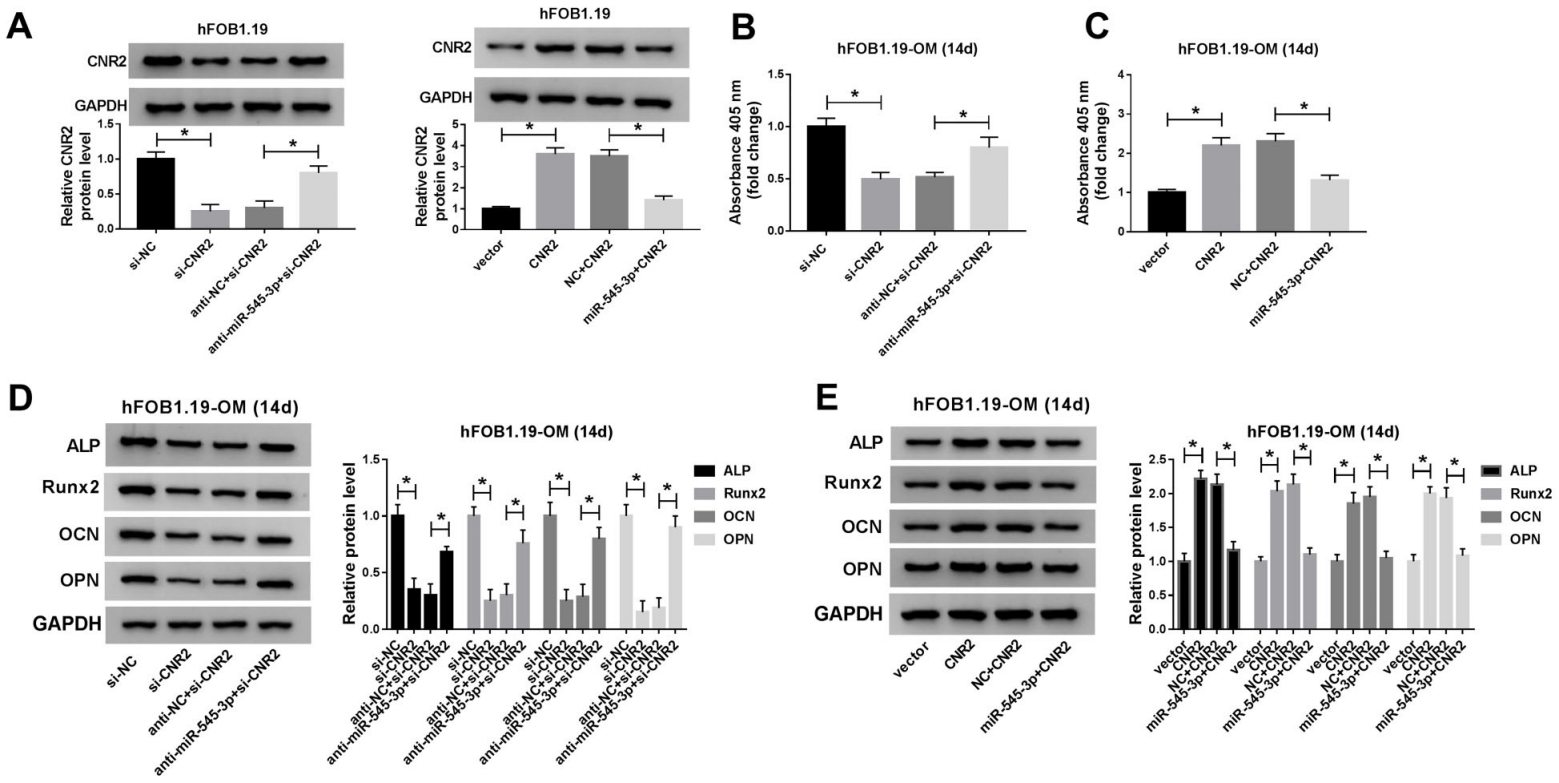
MiR-545-3p inversed the effect of CNR2 on osteogenic differentiation. **A**, The protein level of CNR2 was measured in hFOB1.19 cells transfected with si-NC (negative control), si-CNR2, anti-NC+si-CNR2, or anti-miR-545-3p+si-CNR2. Additionally, CNR2 expression was detected in hFOB1.19 cells introduced with vector, CNR2, NC+CNR2, or miR-545-3p+CNR2. **B**, ALP activity was determined in osteogenic differentiated hFOB1.19 cells transfected with si-CNR2 and/or anti-miR-545-3p. **C**, After introduction with CNR2 and/or miR-545-3p, ALP activity was evaluated in osteogenic differentiated hFOB1.19 cells. **D**, Protein levels of ALP, Runx2, osteocalcin (OCN), and osteopontin (OPN) were measured in osteogenic differentiated hFOB1.19 cells following CNR2 down-regulation and/or miR-545-3p down-regulation. **E**, Expression of ALP, Runx2, OCN, and OPN was detected in osteogenic differentiated hFOB1.19 cells following CNR2 overexpression and/or miR-545-3p overexpression. Data are reported as means±SD of three independent experiments. *P<0.05 (ANOVA).

The decreased expression of CNR2 caused by knockdown of CNR2 and the increased expression of CNR2 induced by overexpression of CNR2 were reversed after transfection with anti-miR-545-3p or miR-545-3p in hFOB1.19 cells, respectively (Figure 8A). Depletion of CNR2 prominently suppressed ALP activity, while suppression of miR-545-3p reversed this effect (Figure 8B). Also, overexpression of CNR2 and miR-545-3p weakened the stimulatory impact of CNR2 upregulation on ALP activity (Figure 8C). Moreover, knockdown of CNR2 significantly reduced the expression of ALP, Runx2, OCN, and OPN, whereas the effect was recovered after reintroduction of anti-miR-545-3p (Figure 8D). Besides, upregulation of CNR2 significantly increased the expression of ALP, Runx2, OCN, and OPN, which were abrogated by upregulating miR-545-3p (Figure 8E). These data indicated that miR-545-3p reversed the effect of CNR2 on osteogenic differentiation in hFOB1.19 cells.

## Discussion

Osteoblast-induced bone formation plays a vital role in bone turnover (21). During bone remodeling, a series of markers of bone turnover are released (22). Runx2 binds to osteoblast-specific cis-acting element 2 to function as a key transcriptional regulator for osteoblast differentiation (23). Osteoblasts produce a variety of extracellular matrix proteins, including ALP, collagen type I, OCN, and OPN (24). Inducing osteogenic differentiation has become a vital therapeutic strategy for bone loss-related diseases (25).

Accumulating evidence has demonstrated that lncRNAs participate in bone remodeling to regulate metabolic bone disease, such as osteoporosis (26). For example, Yu et al. (27) reported that lncRNA PCAT1 positively regulated osteoblast differentiation in human adipose-derived stem cells (hADSCs) via binding to miR-145-5p and increasing TLR4 expression. Xiao et al. (28) discovered that MALAT1 accelerated osteogenic differentiation in human aortic valve interstitial cells (hAVICs) by modulating the miR-204/Smad4 axis. Moreover, Yu et al. (29) revealed that TUG1 facilitated osteoblast differentiation in human aortic valves via sponging miR-204-5p and increasing Runx2 expression. The findings of this study were consistent with previous studies. TUG1 promoted osteoblast proliferation and differentiation.

Investigations have corroborated that lncRNAs could function as competing endogenous RNAs (ceRNAs) to down-regulate miRNAs (14). In the current study, we verified that TUG1 was a sponge of miR-545-3p. Furthermore, miR-545-3p acted as a modulator in a variety of diseases. For example, miR-545-3p targeted MT1M to facilitate the progression of human hepatocellular carcinoma (30). Li et al. (15) reported that SP1-triggered miR-545-3p suppressed the expression of osteogenic differentiation markers and facilitated apoptosis in differentiated MC3T3-E1 cells through inactivation of the LRP5-induced Wnt/β-catenin signaling pathway, thereby inhibiting osteoblast differentiation. Similar to previous research, miR-545-3p expression was decreased in a time-dependent manner in hFOB1.19 cells undergoing osteogenic differentiation. In addition, TUG1 inversed the inhibitory effect of miR-545-3p on osteogenic differentiation, suggesting that TUG1 regulated osteogenic differentiation via sponging miR-545-3p.

Additionally, our research demonstrated that CNR2 was a target of miR-545-3p. A previous report suggested that deficiency of CNR2 expedited age-related bone loss in mice and CNR2 was related to low bone mineral density in females (17). Besides, the absence of CNR1 and CNR2 receptors suppressed osteoclasts, thereby preventing age-related bone loss (31). Furthermore, recent studies have verified that miRNAs could repress the expression of target genes by binding to their mRNAs (32). Xu et al. (33) disclosed that microRNA-187-3p clearly restrained osteogenic differentiation of hFOB1.19 cells via targeting CNR2. In the current study, CNR2 expression was significantly increased in osteogenic differentiated hFOB1.19 cells. Furthermore, mechanism analysis validated that miR-545-3p hindered osteogenic differentiation by targeting CNR2.

In conclusion, TUG1 facilitated the proliferation of osteogenic precursor cells (hFOB1.19 cells). Also, TUG1 sponged miR-545-3p to promote osteogenic differentiation of hFOB1.19 cells by upregulating CNR2. Therefore, our findings provided new insights into the molecular mechanisms of bone metabolism. *In vivo* experiments will support this conclusion, but we had no conditions to conduct *in vivo* assays, which is a limitation of this research.

## References

[1] Feng X, McDonald JM (2011). Disorders of bone remodeling. Annu Rev Pathol.

[2] Charles JF, Aliprantis AO (2014). Osteoclasts: more than ‘bone eaters'. Trends Mol Med.

[3] Rachner TD, Khosla S, Hofbauer LC (2011). Osteoporosis: now and the future. Lancet.

[4] Marie PJ (2013). Targeting integrins to promote bone formation and repair. Nat Rev Endocrinol.

[5] Gibb EA, Brown CJ, Lam WL (2011). The functional role of long non-coding RNA in human carcinomas. Mol Cancer.

[6] Zhou H, Sun L, Wan F (2019). Molecular mechanisms of TUG1 in the proliferation, apoptosis, migration and invasion of cancer cells. Oncol Lett.

[7] Wang Y, Liu G, Ren L, Wang K, Liu A (2019). Long non-coding RNA TUG1 recruits miR29c3p from its target gene RGS1 to promote proliferation and metastasis of melanoma cells. Int J Oncol.

[8] Xie C, Chen B, Wu B, Guo J, Cao Y (2018). LncRNA TUG1 promotes cell proliferation and suppresses apoptosis in osteosarcoma by regulating miR-212-3p/FOXA1 axis. Biomed Pharmacother.

[9] Tang T, Cheng Y, She Q, Jiang Y, Chen Y, Yang W (2018). Long non-coding RNA TUG1 sponges miR-197 to enhance cisplatin sensitivity in triple negative breast cancer. Biomed Pharmacother.

[10] Liu SC, Sun QZ, Qiao XF, Li XG, Yang JH, Wang TQ (2019). LncRNA TUG1 influences osteoblast proliferation and differentiation through the Wnt/beta-catenin signaling pathway. Eur Rev Med Pharmacol Sci.

[11] Lian JB, Stein GS, van Wijnen AJ, Stein JL, Hassan MQ, Gaur T (2012). MicroRNA control of bone formation and homeostasis. Nat Rev Endocrinol.

[12] Lu XD, Han WX, Liu YX (2019). Suppression of miR-451a accelerates osteogenic differentiation and inhibits bone loss via Bmp6 signaling during osteoporosis. Biomed Pharmacother.

[13] Li Y, Feng C, Gao M, Jin M, Liu T, Yuan Y (2019). MicroRNA-92b-5p modulates melatonin-mediated osteogenic differentiation of bone marrow mesenchymal stem cells by targeting ICAM-1. J Cell Mol Med.

[14] Chan JJ, Tay Y (2018). Noncoding RNA: RNA regulatory networks in cancer. Int J Mol Sci.

[15] Li L, Qiu X, Sun Y, Zhang N, Wang L (2019). SP1-stimulated miR-545-3p inhibits osteogenesis via targeting LRP5-activated Wnt/beta-catenin signaling. Biochem Biophys Res Commun.

[16] Marino S, Idris AI (2017). Emerging therapeutic targets in cancer induced bone disease: a focus on the peripheral type 2 cannabinoid receptor. Pharmacol Res.

[17] Bab I, Zimmer A (2008). Cannabinoid receptors and the regulation of bone mass. Br J Pharmacol.

[18] Pertwee RG (2001). Cannabinoid receptors and pain. Prog Neurobiol.

[19] Wang J, Xu Y, Zhu L, Zou Y, Kong W, Dong B (2018). Cannabinoid receptor 2 as a novel target for promotion of renal cell carcinoma prognosis and progression. J Cancer Res Clin Oncol.

[20] Wang B, Lian K, Li J, Mei G (2018). Restoration of osteogenic differentiation by overexpression of cannabinoid receptor 2 in bone marrow mesenchymal stem cells isolated from osteoporotic patients. Exp Ther Med.

[21] Dirckx N, Moorer MC, Clemens TL, Riddle RC (2019). The role of osteoblasts in energy homeostasis. Nat Rev Endocrinol.

[22] Pagani F, Francucci CM, Moro L (2005). Markers of bone turnover: biochemical and clinical perspectives. J Endocrinol Invest.

[23] Ziros PG, Basdra EK, Papavassiliou AG (2008). Runx2: of bone and stretch. Int J Biochem Cell Biol.

[24] Alford AI, Kozloff KM, Hankenson KD (2015). Extracellular matrix networks in bone remodeling. Int J Biochem Cell Biol.

[25] Valenti MT, Dalle Carbonare L, Mottes M (2016). Osteogenic differentiation in healthy and pathological conditions. Int J Mol Sci.

[26] Wu QY, Li X, Miao ZN, Ye JX, Wang B, Zhang F (2018). Long non-coding RNAs: a new regulatory code for osteoporosis. Front Endocrinol (Lausanne).

[27] Yu L, Qu H, Yu Y, Li W, Zhao Y, Qiu G (2018). LncRNA-PCAT1 targeting miR-145-5p promotes TLR4-associated osteogenic differentiation of adipose-derived stem cells. J Cell Mol Med.

[28] Xiao X, Zhou T, Guo S, Guo C, Zhang Q, Dong N (2017). LncRNA MALAT1 sponges miR-204 to promote osteoblast differentiation of human aortic valve interstitial cells through up-regulating Smad4. Int J Cardiol.

[29] Yu C, Li L, Xie F, Guo S, Liu F, Dong N (2018). LncRNA TUG1 sponges miR-204-5p to promote osteoblast differentiation through upregulating Runx2 in aortic valve calcification. Cardiovasc Res.

[30] Changjun L, Feizhou H, Dezhen P, Zhao L, Xianhai M (2018). MiR-545-3p/MT1M axis regulates cell proliferation, invasion and migration in hepatocellular carcinoma. Biomed Pharmacother.

[31] Sophocleous A, Marino S, Kabir D, Ralston SH, Idris AI (2017). Combined deficiency of the Cnr1 and Cnr2 receptors protects against age-related bone loss by osteoclast inhibition. Aging Cell.

[32] Garofalo M, Croce CM (2011). microRNAs: master regulators as potential therapeutics in cancer. Annu Rev Pharmacol Toxicol.

[33] Xu A, Yang Y, Shao Y, Wu M, Sun Y (2019). Inhibiting effect of microRNA-187-3p on osteogenic differentiation of osteoblast precursor cells by suppressing cannabinoid receptor type 2. Differentiation.

